# Domestication history reveals multiple genetic improvements of Chinese bayberry cultivars

**DOI:** 10.1093/hr/uhac126

**Published:** 2022-05-30

**Authors:** Junke Li, Jun Chen, Luxian Liu, Nan Chen, Xian Li, Kenneth M Cameron, Chengxin Fu, Pan Li

**Affiliations:** Laboratory of Systematic & Evolutionary Botany and Biodiversity, College of Life Sciences, Zhejiang University, Hangzhou 310058, China; Laboratory of Systematic & Evolutionary Botany and Biodiversity, College of Life Sciences, Zhejiang University, Hangzhou 310058, China; Laboratory of Plant Germplasm and Genetic Engineering, School of Life Sciences, Henan University, Kaifeng 475000, China; Laboratory of Systematic & Evolutionary Botany and Biodiversity, College of Life Sciences, Zhejiang University, Hangzhou 310058, China; Zhejiang Provincial Key Laboratory of Horticultural Plant Integrative Biology, Zhejiang University, Hangzhou 31058, China; Department of Botany, University of Wisconsin, Madison, WI 53706, USA; Laboratory of Systematic & Evolutionary Botany and Biodiversity, College of Life Sciences, Zhejiang University, Hangzhou 310058, China; Laboratory of Systematic & Evolutionary Botany and Biodiversity, College of Life Sciences, Zhejiang University, Hangzhou 310058, China

Dear Editor,

The Chinese bayberry (*Morella rubra* Lour., 2*n* = 2*x* = 16) is an evergreen fruit tree native to southern China and the only domesticated species in family Myricaceae [1]. Today *M. rubra* is widely cultivated in subtropical regions of China and has become an economically important fresh fruit with >300 varieties and an annual production of 1.5 million tons [2, 3]. The aim of the current study was to reveal the domestication history of *M. rubra* using restriction site-associated DNA sequencing (RAD-seq) data. Seventy-eight individuals were sampled, including 44 landraces and improved cultivated varieties, 19 wild individuals from the natural distribution ranges in southern China, 14 from five other *Morella* species, and 1 from *Comptonia*.

A maximum likelihood phylogenetic tree was generated using 67 064 single-nucleotide polymorphisms (SNPs) with linkage disequilibrium (LD) pruned and rooted with *Comptonia peregrina* (Fig. 1a). Four wild individuals from southwestern China, two from Yunnan province (YNML and YNDW), one from Chongqing (CQNC), and one from Guizhou province (GZLS), were at the basal position of the *M. rubra* clade, indicating a southwest origin of *M. rubra*. Moreover, four wild individuals from the east coast (ZJGT, ZJWY, ZJDP from Zhejiang province, and FJNP from Fujian province) formed the closest sister clade to all cultivated varieties, which suggested that the cultivars are genetically closer to these wild individuals. The primary domestication most likely took place on the east coast of China. All cultivated bayberries formed a monophyletic group that consisted of four lineages: (1) the landraces (PL); (2) the cultivar ‘Dongkui’ (DK, first developed in Zhejiang and now the most popular cultivar planted over China) together with two cultivars, ‘Langdangzi’ and ‘Ding’ao’, from Jiangsu and Zhejiang; (3) the cultivar ‘Biqi’ (BQ) and derived variants that were also developed in Zhejiang; and (4) other cultivars in Zhejiang and Jiangsu (FH). Five wild individuals were mixed in the cultivated group (GZYS, JXGZ, FJXC, GXNIN, and JXNC). They could have escaped recently, and were removed from the demographic inference. This suggests that all extant Chinese bayberry cultivars resulted from a single domestication event.

**Figure 1:**
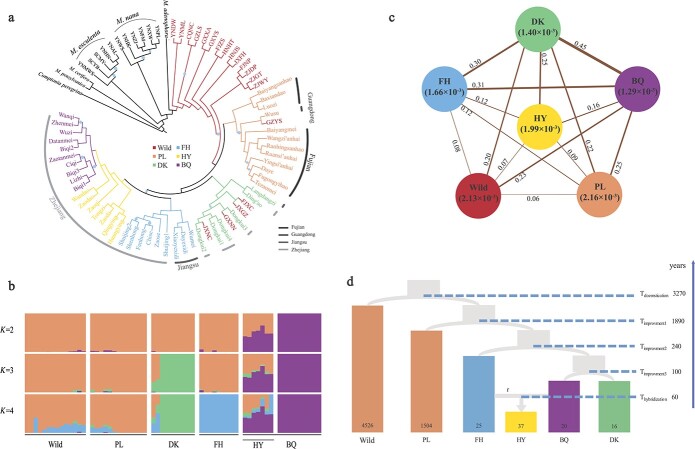
Phylogeny, population genetics, and demographic history of wild and domestic Chinese bayberry. **a** Maximum likelihood (ML) tree based on 67 064 SNPs. Bootstrap values <90 are marked with blue circles on the branch, the size of circle represents the size of value. Bars in different gray scales indicated Fujian, Guangdong, Jiangsu, and Zhejiang origins. **b** Individual ancestry coefficients of RAD accessions estimated by ADMIXTURE. The orders of these individuals on the *x* axis are consistent with those for the ML tree. **c** Nucleotide diversity (*π*) and population divergence (*F*_ST_) across the six groups. Line thickness is proportional to *F*_ST_. **d** Graphical summary of the best fitting demographic model inferred by fastsimcoal2. Estimated divergence time and effective population size are detailed in Table 1.

ADMIXTURE analysis was conducted on all *M. rubra* individuals and from *K* = 2 to 4, and the lineage of BQ, DK and FH showed different ancestry components (Fig. 1b). The seven individuals in the BQ lineage comprise admixed components and possibly were results of the hybridization (named ‘HY’ hereafter) of BQ and other lineages. The lineage of PL shared the same ancestry component with the wild group, partly admixed with FH when *K* = 4 as well. Summary statistics of population genetics were calculated for six groups (wild, PL, DK, BQ, HY, and FH). The wild and PL groups harbored the largest genetic diversity, *π* = 0.0021 for both. As expected, cultivars had the lowest genetic diversity, *π* = 0.0013 in BQ and *π* = 0.0014 in DK; FH had a slightly higher value, *π* = 0.0017, possibly because it contained several different cultivars. The HY group had a genetic diversity level close to the wild and landrace groups (*π* = 0.002, Fig. 1c). The greatest genetic divergence was found between cultivars, with *F*_ST_ = 0.45 between BQ and DK, 0.31 between BQ and FH, and 0.3 between DK and FH. The lowest genetic divergence was observed between the wild, PL, and HY groups, with *F*_ST_ varying from 0.06 to 0.09.

Based on coalescent simulation of six groups (wild, PL, FH, BQ, DK, and HY), the best point estimates of parameters of the best-supported model gave us more clues about the domestication history of *M. rubra*. The primary domestication event that separated PL from the wild bayberry most likely occurred ~3300 years ago [95% confidence interval (CI) 2446–8573; Fig. 1d and Table 1], and was followed by an improvement event of PL to the ancestor of all cultivars ~1900 years ago (95% CI 480–2800). Our results also suggested that various cultivars may have flourished ~240 years ago (95% CI 110–1730), whereas two of today’s most popular cultivars, ‘Dongkui’ and BQ (‘Biqi’ and its variants), may have been developed from a common cultivar ~100 years ago (95% CI 60–540). The model comparison showed that potential hybridization between BQ and FH could have occurred ~60 years ago (95% CI 10–180), probably as a result of modern breeding. The wild Chinese bayberry possessed the largest effective population size, *N*_e_ ~4500 (95% CI 3242–11 780). The landraces (PL) had a value only one-third of that, ~1500 (95% CI 390–2244), while cultivars and the hybrid harbored extremely small *N*_e_, ranging from 16 to 37 (95% CI 10–120).

**Table 1:** Inferred demographic parameters of the best-fitting demographic model (see Fig. 1d).

**Parameter**	**Best point estimation**	**95% CI**
Effective population size, *N*		
*N*_Wild_	4526	3242–11 780
*N*_PL_	1504	390–2244
*N*_FH_	25	10–103
*N*_DK_	16	10–89
*N*_BQ_	20	10–72
*N*_HY_	37	10–122
Admix proportion, *r*	0.54397	0.22–0.68
Divergence time, *T* (years)		
*T*_hybridization_	60	10–180
*T*_improvement3_	100	60–540
*T*_improvement2_	240	110–1730
*T*_improvement1_	1890	480–2800
*T*_domestication_	3270	2450–8570

Compared with the previous study of Liu *et al*. (2015) [4], our study focused mainly on the origination and domestication history of *M. rubra*. It suggests that wild *M. rubra* may have originated in southwestern China, e.g. Yunnan, Guizhou, or Sichuan. It then spread to the east coast, where, most likely in Zhejiang or Fujian, a single domestication event of wild *M. rubra* took place. The domestication could be rather primitive, possibly in a sort of ‘self-domestication’ way whereby twigs of wild trees were propagated by the householder, as is still done in some villages today. Thus, hardly any genetic diversity loss was observed between the wild group and the landraces. Though earlier than what we report here, a great number of pollens of the *Morella* genus have been discovered in the excavation of the Neolithic site at Hemudu [7000 calibrated years before the present (cal BP)] in Zhejiang province [5, 6], which gives a hint that such primitive domestication activity could be a possible explanation.

On the contrary, the major genetic differentiation was observed between two elite cultivars DK and BQ (*F*_ST_ = 0.45), in which a significant reduction of genetic diversity was also found. This suggests that improvement events play a more crucial role in the development of selected traits of extant cultivars. The first improvement, which gave rise to the common ancestor of all cultivars, took place 1890 (480–2800) years ago, approximately corresponding to the Western Han Dynasty or even earlier. The fruits of Chinese bayberry were discovered in the array of funeral objects in ‘Mawangdui’ tombs, the most famous Han Dynasty tombs (2200 cal BP). The first documentary about the cultivation of Chinese bayberry was also made in the Western Han Dynasty by Chen (1996) [7]. The second improvement occurred rather recently, ~240 (110–1730) years ago, when various cultivars appeared. A great leap was made in horticultural and agricultural techniques in the Song, Yuan, and Ming Dynasties and grafting was commonly applied to facilitate the breeding of ornamental plants and fruit trees, e.g. *Paeonia suffruticosa*, *Prunus mume*, *Prunus persica*, *Prunus salicina*, and *M. rubra* [8]. By the Southern Song Dynasty (~743–895 BP) Chinese bayberry had been widely cultivated and grafted in regions nowadays around Zhejiang Province, and multiple elite cultivars had spread to other parts of southern China (Chen *et al.*, 2004). The two most popular cultivars, BQ and DK, were derived from the same ancestor and developed from a third independent improvement ~100 (60–540) years ago, approximately in the Qing Dynasty. A recent study of the genomic DNA footprint of 14 DK individuals suggests that they were all propagated from one mother tree ~180 years old that lives in Huangyan, Zhejiang province [9]. The local chronicles of Zhejiang (浙江通志) published in the Qing Dynasty described the breeding traits of DK and BQ: ‘the fruit in a color dark like water chestnut, made up of tiny and dense finger-like sections and tastes sweet’, quite similar to those of the modern cultivars. However, it is a bit surprising that the hybridization event between BQ and some other cultivars (FH) was found to be so recent, ~60 (10–180) years ago. In fact, due to the maintenance of cultivars (female trees) by asexual propagation, little documentation was found on the improvement of cultivars by crossbreeding through thousands of years of cultivation history in China [10]. Recent research of Jiao *et al*. (2013) [11] reported that pollens of a mutated branch of cultivars allowed crossing between cultivars. Modern hybridization results in more variants of BQ, e.g. ‘Wandao’, the fruits of which mature 15–20 days later than BQ on average.

On summary our study has for the first time clarified the domestication history of *M. rubra* and provided valuable information for future research and breeding activities based on genetic diversity.

## Acknowledgements

This work was supported by the National Natural Science Foundation of China (grants 31970225, 31972946, and 31461123001) and the Zhejiang Provincial Natural Science Foundation (grant LY19C030007). We sincerely thank Yonghua Zhang and Luxian Liu for their great help with collecting plant materials.

## Author contributions

P.L., C.F., J.L., and J.C. conceived the study; L.L., N.C., and P.L. contributed to the sampling; L.L. and N.C. performed the experiments; J.L., J.C., and P.L. analyzed the data. The manuscript was written by J.L. and J.C. and revised by K.M.C., P.L., C.F., and X.L.

## Data availability

The raw resequencing data have been deposited in the Sequence Read Archive of the National Center for Biotechnology Information (NCBI) with BioProject accession number PRJNA606201. The detailed supplementary material and sampling information are available at https://github.com/JUNKELII/RAD/blob/main/supplementary.docx.
